# Daily Medical Liaison Is Associated with Reduced Length of Stay and Complications in Selected Patients Admitted to a Regional Vascular Surgery Service

**DOI:** 10.3390/geriatrics5040078

**Published:** 2020-10-17

**Authors:** Emma Mitchell, Roisin Coary, Paul White, Emily Farrow, Amy Crees, William Beedham, Mark Devine, Rebecca Winterborn, David Shipway

**Affiliations:** 1 Department of Medicine for Older People, North Bristol NHS Foundation Trust, Bristol BS10 5NB, UK; emma.mitchell2@nbt.nhs.uk (E.M.); Emily.Farrow@nbt.nhs.uk (E.F.); amycrees@nhs.net (A.C.); Mark.Devine@nbt.nhs.uk (M.D.); 2 Department of Medicine for the Elderly, St James’s Hospital, P.O. Box 580 Dublin, Ireland; coaryr@tcd.ie; 3 Applied Statistics Group, Faculty of Environment and Technology, University of the West of England, Bristol BS16 1QY, UK; paul.white@uwe.ac.uk; 4 Birmingham Medical School, University of Birmingham, Birmingham B15 2TT, UK; WXB544@student.bham.ac.uk; 5 Department of Vascular Surgery, North Bristol NHS Foundation Trust, Bristol BS10 5NB, UK; Rebecca.Winterborn@nbt.nhs.uk; 6 Honorary Senior Clinical Lecturer, Department of Population Health Sciences, University of Bristol, Bristol BS8 1QU, UK

**Keywords:** surgery, ageing, perioperative medicine, postoperative complications

## Abstract

Older adults undergoing vascular surgery are particularly vulnerable to adverse outcomes by virtue of their vascular risk factors and medical comorbidities. This study aimed to determine the impact of daily medical liaison for patients aged 65 years and older admitted to a regional vascular surgery centre. This was a descriptive before-and-after study concerning 375 patients. The primary outcome measure was length of stay (LOS). Following intervention, we identified a reduction in mean LOS in the sample from 10.75 to 7.95 days (*p* = 0.635, 95% Confidence Interval [CI] 0–5 days) with a statistically significant reduction in mean LOS for “stranded” patients admitted for more than seven days (mean 7.84 days reduction, *p* = 0.025, 95% CI for mean difference, 1.5 to 14 days). These patients did not display elevated 30-day readmission rates (12/60 to 8/72, *p* = 0.156, 95% CI −3% to 21%). A non-significant reduction in postoperative complications was seen in all patients in the post-intervention cohort (1.09 to 0.86 per person, *p* = 0.181, 95% CI −0.11 to 0.56), reaching statistical significance in emergency vascular admissions (1.81 to 0.97 complications per person, *p* = 0.01, mean difference = 0.84, 95% CI 0.21–1.46). This study demonstrated reduced LOS and complications in selected older patients admitted under vascular surgery after the introduction of a daily medical liaison model. These data are amongst the first to reproduce randomised controlled trial findings in a non-trial setting. Subgroup analysis indicates that patients admitted with acute pathology and those with long LOS may benefit most from medical liaison where resources are finite.

## 1. Introduction

The proportion of older people undergoing surgery is increasing faster than the rate of population ageing [1,2]. This is representative of advances in surgical and anaesthetic techniques. However, it is well established that older patients are more susceptible to adverse outcomes and those undergoing vascular surgery are a particularly vulnerable, high-risk group [2,3]. This frequently reflects the presence of multiple comorbidities (multimorbidity) including hypertension, diabetes, ischaemic heart disease and additional lifestyle risk factors such as smoking. Distinct from older age and multimorbidity is frailty, a syndrome of vulnerability to minor stressors as a consequence of accumulated deficits over an individual’s lifetime leading to reduced physiological reserve. Frailty independently predicts adverse outcomes after major vascular surgery [4].

Associated with the increasing volumes of older adults and/or those with multimorbidity and frailty presenting to vascular centres, is the increased burden of postoperative complications. Whilst older adults can have good outcomes from vascular surgery, the postoperative period is often protracted and syndromes such as delirium commonly lead to functional decline and increased dependency. Whilst patient awareness of these risks are of fundamental importance to informed consent and shared decision making, detailed risk assessment alone cannot be expected to reduce the risk of adverse outcome. A solution to this may lie in Comprehensive Geriatric Assessment (CGA). CGA is an evidence-based tool utilised by geriatricians to improve clinical outcomes for older people in a variety of clinical settings [5]. CGA has been defined within a Cochrane systematic review as a “*multi-dimensional, multi-disciplinary diagnostic and therapeutic process, conducted to determine the medical, mental, and functional problems of older people with frailty, so that a co-ordinated and integrated plan for treatment and follow-up can be developed*” [6].

Perioperative Comprehensive Geriatric Assessment (CGA) has been studied in recent years in a range of surgical settings, and has been associated with improved outcomes in orthopaedic, gastrointestinal and urological surgery [7,8,9]. More recently, CGA has been evaluated in the context of older patients undergoing vascular surgery using randomised trial study design [10]. In this setting, CGA has been shown to reduce length of stay (LOS), reduced complications and reduced discharge dependency [10].

In our centre, medical support for surgical teams was historically provided by ad hoc reactive review provided on demand by the duty medical registrar, or by direct inpatient referral to medical specialties. In this service development, we aimed to determine whether the CGA-derived service intervention evaluated in the randomised trial by Partridge et al. could be replicated in a non-trial, service development setting in our regional tertiary vascular centre. Our primary outcome measure was reduction in LOS. Our secondary outcome measure was reduction in the number of postoperative complications.

## 2. Materials and Methods

The study was conducted at an 800-bedded hospital providing tertiary-level care for vascular surgery patients. This was a single-centre, non-randomised, before-and-after study design comparing pre-existing conventional practice with a model of care previously shown to be effective in other surgical settings [9]. This comprised daily senior-led (registrar/consultant) medical liaison reviews provided by geriatric medicine physicians. A summary of the acute presenting issues, underlying comorbidities and relevant psychosocial factors, such as history of cognitive impairment, would be explored. With this information, a problem list was created, inclusive of perioperative risk stratification and the identification of patient-specific factors that might make an individual susceptible to postoperative complications. The plan and follow-up were executed by the vascular surgery team with our liaison service providing repeated reviews where necessary. This service was provided within normal working hours (08:00–17:00, Monday to Friday). Patient identification was triggered through direct liaison with the vascular surgery team and daily attendance to the vascular surgery ward to case-find. Patients were seen either in a preoperative capacity (often aiding surgical decision making) or, in the immediate postoperative period.

Electronic records and patient case notes were analysed for all patients aged 65 years and older admitted for one or more nights. Notes were analysed retrospectively during two three-month periods across two consecutive years (January–March 2017 and 2018) to allow pre- and post-intervention analysis.

Data collection was conducted by a team of doctors and one medical student working within the Department of Medicine for Older People. Patient demographics were recorded including sex, age, admission type (emergency/elective), source of admission (home/other hospital/care home), operation type, comorbidities and frailty scores. Comorbidities were recorded using the Charlson comorbidity index [11]. Frailty was recorded using the Clinical Frailty Scale (CFS) [12]. Outcome variables included LOS and, for patients undergoing surgery, the number of complications suffered. Complications were recorded according to actual number and using guidance from The Clavien–Dindo system [13]. Other information recorded included admission to intensive care, 30-day readmission rates and inpatient mortality.

Statistical analysis was performed using SPSS Statistics 23. Between-group differences were analysed using correlation analysis, chi-square test of association, odds ratios, two-sample independent tests, and Kaplan-Meier analysis as appropriate. We determined that for moderate but clinically important effects, a sample size of 150 patients in each of the pre- and post-intervention cohorts would provide this study with a power in excess of 80% for detecting a standardised difference of 0.35 standard deviations (SD), a correlation of *r* = 0.25 and for detecting an odds ratio of 2:1 over a range of proportions likely to be encountered in practice.

## 3. Results

### 3.1. Study Population Characteristics

In the pre-intervention group, 171 patient case-notes were reviewed with 205 in the post-intervention group. Average age was 76 (range 65–95) pre-intervention and 77 (range 65–97) post-intervention (mean difference = −0.4, *p* = 0.607, 95% CI −1.4 to 1.8). Average CFS scores were 4.08 pre-intervention and 4.27 post-intervention (*p* = 0.058) corresponding with “vulnerability” rather than frailty, which is classed as a CFS score of ≥5. The frequency of admission to intensive care remained stable at 20% following intervention (34/171 pre and 41/204 post-intervention, 95% CI −8.3% to 7.9%), and 30-day readmission rates remained unchanged (22/171 versus 24/204, *p* = 0.746, 95% CI −5.6% to 7.8%). Mortality showed a non-significant reduction of 1.4% (9/171 to 8/204, *p* = 0.534, 95% CI −2.9% to 5.6%). See Table 1 for further patient demographic details.

### 3.2. Length of Stay

Following the implementation of our medical liaison service, overall mean LOS showed a favourable reduction from 10.75 to 7.95 days, which did not reach statistical significance (*p* = 0.635, CI 0 to 5 days). When comparing the impact of our intervention on LOS for patients admitted either acutely or electively, there was no significant reduction seen (*p* = 0.103, 95% CI 0 to 5 days and *p* = 0.890, 95% CI −1 to 0 days respectively).

**Figure 1:**
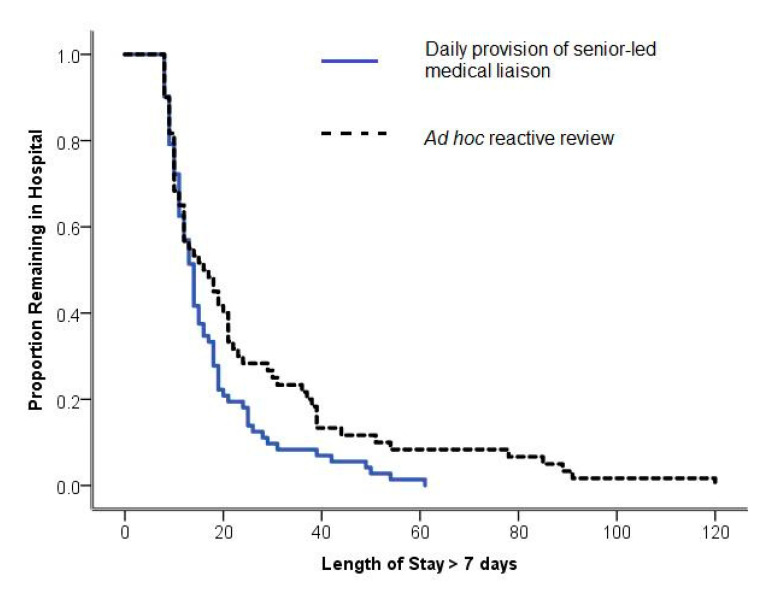
Kaplan-Meier Survival Curve. Reduction in LOS was seen for patients admitted for >7 days (*p* = 0.025, 95% CI for mean difference, 1.5 to 14 days).

Post hoc Kaplan-Meier analysis (Figure 1) and a comparison of means showed a significant reduction from 25.12 to 17.28 days in mean LOS for patients admitted for more than seven days (*p* = 0.025, 95% CI for mean difference 1.5 to 14 days). Closer evaluation of this cohort demonstrated well-matched demographic data; specifically, there were no differences in patient sex (*p* = 0.774), age (*p* = 0.923), type of surgery (elective/acute) (*p* = 0.710) or rates of intensive care admission (*p* = 0.696). Utilising correlation analysis, age did not significantly correlate with LOS either pre-intervention (*r* = −0.11, *p* = 0.171) nor post-intervention (*r* = −0.127, *p* = 0.064). Lastly, for patients admitted for longer than seven days, 30-day readmission rates showed a non-significant reduction from 12/50 (20.0%) to 8/72 (11.1%), (*p* = 0.156, 95% CI −3% to 21%).

### 3.3. Postoperative Complications

The total number of postoperative complications suffered per patient demonstrated a non-significant reduction for all patients following service implementation (1.09 to 0.86 per person, *p* = 0.181, CI −0.11 to 0.56). Complications suffered by patients admitted acutely were more frequent compared to those admitted electively (1.35 versus 0.71 per person, *p* < 0.001, mean difference = 0.64, 95% CI 0.29 to 0.98). When evaluating complication frequency following service implementation in elective patients, there was no significant reduction (0.63 to 0.79 per person, *p* = 0.373, mean difference −0.16, 95% CI −0.60 to 0.19). However, when evaluating complication frequency in the acute patients following service implementation, there was a significant reduction (1.81 to 0.97 per person, *p* = 0.01, mean difference = 0.84, 95% CI 0.21 to 1.46). Correlation analysis found that age did not significantly correlate with frequency of complications either pre-intervention (*r* = −0.069, *p* = 0.405) nor post-intervention (*r* = −0.120, *p* = 0.122).

## 4. Discussion

This study, evaluating the initial impact of our service, has demonstrated that routine, proactive, senior-led medical liaison for older multimorbid patients admitted under vascular surgery can have clinical benefits for selected patients. Firstly, a favourable reduction in mean LOS by 2.8 days was observed, with a significant reduction (7.84 days) in LOS for a subgroup of patients admitted for more than seven days. This was seen without a rise in readmission rates. This time period reflects “stranded” patient status and is an increasingly used metric utilised in the NHS to identify patients with complexity and delayed discharge [14]. Secondly, there was a favourable, albeit non-significant, reduction in complication frequency in all patients following service implementation, with statistically significant reductions seen in patients admitted acutely. This may indicate that patients who gain most from medical liaison are those admitted with acute pathology, and those who sustain a long length of stay. Long length of stay is typically associated with complexity and complications, and it therefore plausible that medical liaison may be of most value in this patient group. Note should be made that the primary outcome measure of reduced length of stay in all patients did not reach statistical significance. This may reflect patient characteristics such as frailty, or that the intervention was insufficiently intense to be able to influence outcome in this study. Furthermore, neither LOS nor complication frequency was associated with age, supporting the notion that age alone should not be used to inform surgical decision making.

The findings of this study are similar to reports assessing the impact of geriatric liaison in orthopaedic, urological and gastrointestinal surgery. CGA is the united methodology behind these studies, and it has been proposed that clinical benefits may be achieved through prompt recognition and management of postoperative complications, and a proactive approach to postoperative goal-setting and discharge planning [7,8,9]. As Partridge et al. established in their randomised controlled trial concerning patients scheduled for vascular surgery, CGA can provide an opportunity to recognise previously undiagnosed pathology across several domains including delirium and comorbidity [10].

This study included all patients aged 65 years and older admitted for one or more nights; this enhances the generalisability of our results. Few demographic differences were seen between the pre-intervention and post-intervention groups and none reached statistical significance. Notably, the potential benefits of length of stay reduction were not offset by increased readmission rates. However, note should be made that the average CFS frailty score in both groups was, respectively, 4.08 and 4.27. A typical threshold of CFS ≥ 5 is accepted to indicate frailty, and it is therefore possible that many patients in our study were insufficiently frail to benefit from CGA. This may explain why trend reductions not reaching statistical significance were seen in the primary outcome measures, and only found in sub-group analysis.

Furthermore, this study has important limitations which may have introduced uncontrolled bias. These include retrospective and single-centre study design, where data extraction from case notes was performed in part by clinicians who participated in clinical reviews. Furthermore, there was a focus on service development with reliance on clinical records to capture clinical details. To minimise the risk of incomplete data, electronic records such as discharge letters were cross-referenced with the clinical notes to enhance accuracy. It was noted that discharge letters did not often comprehensively summarise key medical issues, potentially leading to the underreporting of complications. Another limitation was that the service was delivered during normal working hours (08:00–17:00, Monday to Friday) and, therefore, results must be interpreted with the understanding that outside of these hours, a reactive method was adopted which was reliant on acute services, such as ad hoc referral to the duty medical registrar.

## 5. Conclusions

In conclusion, these data indicate that existing RCT results demonstrating the benefits of proactive medical liaison for complex older patients undergoing vascular surgery may be partially reproduced in a service development setting with modest resource allocation. The study indicates that daily medical liaison can generate some clinically significant reductions in length of stay, complication frequency and readmission rates in selected patients. These effects reached statistical significance in patients admitted acutely and in those with longer lengths of stay. These clinical and economic advantages for selected patients indicate that long-term investment in medical liaison for patients admitted acutely under vascular surgery, or those sustaining long LOS, may be justified. Where medical liaison resource is limited, we advocate the deployment of available resources to target vascular surgery patients with long LOS and those admitted with acute vascular pathology.

## Figures and Tables

**Table 1:** Demographics of adults aged 65 years and older admitted under vascular surgery in the pre- and post-intervention study groups. *Numbers are percentages with raw values in parentheses unless otherwise stated*.

	Pre-Intervention (n = 171)	Post-Intervention (n = 204)	*p* Value
**Sex**			0.622
Male	64% (109)	66% (135)	
Female	36% (62)	34% (69)	
**Admission Type**			0.612
Emergency	47% (81)	50% (102)	
Elective	53% (90)	50% (102)	
**Source of Admission**			0.422
Home	79% (135)	79% (162)	
Other Hospital	16% (27)	18% (36)	
Care Home	5% (9)	3% (6)	
**Operation Type**			0.638
Carotid Endarterectomy	11% (18)	9% (19)	
Angiography/Embolectomy	25% (42)	30% (61)	
Bypass	16% (28)	10% (20)	
Amputation of Limb	11% (18)	9% (19)	
Endovascular AAA	13% (22)	13% (26)	
Open AAA	4% (7)	6% (11)	
Conservative Management	13% (23)	16% (33)	
Other	7% (13)	7% (15)	
**Charlson Comorbidity Index**			0.684
Average Score	6.16	6.29	
**Clinical Frailty Score**			0.058
Average Score	4.08	4.27	

## References

[1] Etzioni D., Liu J., O’Connell J. (2019). Elderly patients in surgical workloads: A population-based analysis. Am. Surg..

[2] Hamel M., Henderson W., Khuri S., Daley J. (2005). Surgical Outcomes for Patients Aged 80 and Older: Morbidity and Mortality from Major Noncardiac Surgery. J. Am. Geriatr. Soc..

[3] Turrentine F., Wang H., Simpson V., Jones R.S. (2008). Surgical risk factors, morbidity, and mortality in elderly patients. Crit. Rev. Oncol. Hematol..

[4] Wang J., Zou Y., Zhao J., Schneider D., Yang Y., Ma Y., Huang B., Yuan D. (2018). The Impact of Frailty on Outcomes of Elderly Patients After Major Vascular Surgery: A Systematic Review and Meta-analysis. Eur. J. Vasc. Endovasc. Surg..

[5] Ellis G., Whitehead M., Robinson D., O’Neill D., Langhorne P. (2011). Comprehensive geriatric assessment for older adults admitted to hospital: Meta-analysis of randomised controlled trials. BMJ.

[6] Ellis G., Gardner M., Tsiachristas A., Langhorne P., Burke O., Harwood R.H., Conroy S.P., Kircher T., Somme D., Saltvedt I. (2017). Comprehensive geriatric assessment for older adults admitted to hospital. Cochrane Database Syst. Rev..

[7] Braude P., Goodman A., Elias T., Babic-Illman G., Challacombe B., Harari D., Dhesi J. (2016). Evaluation and establishment of a ward-based geriatric liaison service for older urological surgical patients: Proactive care of Older People undergoing Surgery (POPS)-Urology. BJU Int..

[8] Harari D., Hopper A., Dhesi J., Babic-Illman G., Lockwood L., Martin F. (2007). Proactive care of older people undergoing surgery (‘POPS’): Designing, embedding, evaluating and funding a comprehensive geriatric assessment service for older elective surgical patients. Age Ageing.

[9] Shipway D., Koizia L., Winterkorn N., Fertleman M., Ziprin P., Moorthy K. (2018). Embedded geriatric surgical liaison is associated with reduced inpatient length of stay in older patients admitted for gastrointestinal surgery. Future Healthc. J..

[10] Partridge J., Harari D., Martin F., Peacock J.L., Bell R., Mohammed A., Dhesi J.K. (2017). Randomized clinical trial of comprehensive geriatric assessment and optimization in vascular surgery. Br. J. Surg..

[11] Charlson M., Pompei P., Ales K., MacKenzie C.R. (1987). A new method of classifying prognostic comorbidity in longitudinal studies: Development and validation. J. Chronic Dis..

[12] Rockwood K., Mitnitski A. (2007). Frailty in Relation to the Accumulation of Deficits. J. Gerontol. Ser. A Biol. Sci. Med. Sci..

[13] Dindo D., Demartines N., Clavien P. (2004). Classification of Surgical Complications. Ann. Surg..

[14] Improvement.nhs.uk (2020). Reviewing ‘Stranded’ Patients in Hospital—What Are Patients Waiting For?. https://improvement.nhs.uk/documents/631/reviewing-stranded-patients-in-hospital-RIG.pdf.

